# PCD Increases Impact Factor and Establishes Statistics Advisory Committee

**DOI:** 10.5888/pcd16.190238

**Published:** 2019-08-15

**Authors:** Leonard Jack

**Figure:**
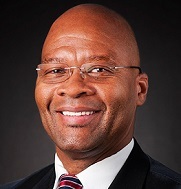
Leonard Jack Jr, PhD, MSc

*Preventing Chronic Disease* (PCD) addresses issues of importance in chronic disease research and practice, issues based not only on external input we receive from experts in the field of public health, but also from our associate editors and editorial board, senior leadership in the National Center for Chronic Disease Prevention and Health Promotion, our dedicated and talented staff, and our readers. The targeted steps we have taken have helped us to recruit the best researchers in the field, including experts who ensure our published articles meet the highest scientific standards, thereby delivering quality content to our growing readership and finding ways to expand the scope of the journal’s expertise. We are excited to see many of the plans we initiated over the past few years come to fruition.

We have been fortunate this year to secure additional expert members for our editorial board and our team of associate editors. These experts add breadth and depth to our content areas of specialization and represent an impressive mix of expertise in geospatial epidemiology, health economics, program planning, implementation evaluation, health systems research, and community trials. They have made enormous contributions to the quality of the science PCD publishes, have acted as mentors in guiding authors through the peer-review process, and have provided valuable input on relevant and timely content areas. In turn, PCD has worked to ensure that our associate editors receive active and useful critiques on manuscripts. Our editorial office has taken steps to carefully refine PCD’s pool of peer reviewers, ensuring that associate editors receive timely and high-quality feedback for authors.

## Increased Impact Factor and Other Metrics of Success

These efforts undoubtedly contributed to the journal’s new impact factor of 2.028, an impressive jump from the 1.862 of the previous year. PCD is also seeing success through increased article downloads and webpage views. Data from 2018 indicate that 54,906 PDF articles were downloaded, and PCD’s website had 998,086 views (1). The journal continues its position in 14th place as one of Google Scholar’s top 20 public health journals but moved up in its Scimago Journal rank from 20th to 18th place out of 139 US journals in the category of public, environmental, and occupational health in the *Science Citation Index Expanded* edition of *Journal of Citation Reports*.

## Formation of the PCD Statistical Advisory Committee

While we appreciate these successes, we continue to look for ways to build on them. A next step — a critical one — is to build the journal’s capacity to assess the quality and rigor of an increasing number of submitted articles that present complex and sophisticated statistics formulas, analyses, and interpretations. We are pleased to announce the formation of the PCD Statistics Advisory Committee (SAC). The goal of SAC is to advance understanding and dissemination of statistical methods and testing in the public health field and to contribute to the growing body of knowledge of statistics that can be applied to understanding what may contribute to decreasing chronic disease and improving the health of individuals across their life span. PCD’s SAC members will be volunteers with training and expertise in statistics and biostatistics who will assess peer-reviewed articles to determine the appropriateness of research and evaluation questions, selection of the study sample, suitability of statistical tests, analysis of data based on research design, distribution of data, and type of variable under examination. SAC members will also assess whether reported findings are accurate and logically aligned with the results presented and discussed in articles. Volunteers selected for SAC will serve a 3- to 4-year renewable term and will be guided by an appointed chair and co-chair. SAC members will provide guidance to peer reviewers on how and what to assess in their reviews of manuscripts that present complex statistical content. Please visit https://wwwdev.cdc.gov/pcd/about_the_journal/Statistics_Advisory_Committee.htm for more information on SAC and it members.

The response to our recruitment efforts for SAC has been tremendous. We are excited about the direction the journal is moving in and by the support and commitment it is receiving from professionals in the field. We hope to continue to position PCD for success, measured not only by the journal’s impact factor and citation, uptake, and outreach data but also by intrinsic qualities such as the positive response and support we receive from other public health professionals. Should you or someone you know be interested in becoming a SAC member, please submit your brief biography via email to pcdeditor@cdc.gov for consideration. PCD will welcome your interest and participation.

## Closing Thoughts

PCD is committed to serving as a valuable resource to promote dialogue among researchers, evaluators, practitioners, and policy makers working in public health worldwide. PCD will continue to take the necessary steps to identify and publish timely content, improve the quality and reach of its publication, draw in the best expertise to publish statistically accurate research that moves the field forward, and provide updates on the journal’s steps in bringing all of this to pass.
